# Aspects of exercise with person-centred guidance influencing the transition to independent exercise: a qualitative interview study among older adults with rheumatoid arthritis

**DOI:** 10.1186/s11556-019-0211-8

**Published:** 2019-04-05

**Authors:** Elvira Lange, Annie Palstam, Inger Gjertsson, Kaisa Mannerkorpi

**Affiliations:** 1 Department of Health and Rehabilitation, Unit of Physiotherapy, Institute of Neuroscience and Physiology, The Sahlgrenska Academy, University of Gothenburg, Box 455, 405 30 Göteborg, Gothenburg, Sweden; 2 University of Gothenburg Center for Person-centred Care, The Sahlgrenska Academy, University of Gothenburg, Gothenburg, Sweden; 3 Department of Clinical Neuroscience, Institute of Neuroscience and Physiology, The Sahlgrenska Academy, University of Gothenburg, Gothenburg, Sweden; 4 Department of Rheumatology and Inflammation Research, Institute of Medicine, The Sahlgrenska Academy, University of Gothenburg, Gothenburg, Sweden

**Keywords:** Rheumatology, Exercise, Person-centered, Person-centred, Patient-centered, Physiotherapy, Physical therapy, Qualitative research

## Abstract

**Background:**

Besides being health enhancing and disease preventing, exercise is also an important part of the management of chronic conditions, including the inflammatory joint disease rheumatoid arthritis (RA). However, older adults with RA present a lower level of physical activity than healthy older adults. The aim of this qualitative study was to explore aspects of participation in moderate- to high-intensity exercise with person-centred guidance influencing the transition to independent exercise for older adults with RA.

**Methods:**

A qualitative interview study was conducted. In-depth interviews with 16 adults with RA aged between 68 and 75 years, who had taken part in the intervention arm of a randomized controlled trial performing moderate- to- high-intensity exercise with person-centred guidance, were analysed using qualitative content analysis.

**Results:**

The analysis resulted in six main categories: A feasible opportunity to adopt exercise, Experiencing positive effects of exercise, Contextual factors affect the experience of exercise, Developing knowledge and thinking, Finding one’s way, and Managing barriers for exercise. The exercise with person-centred guidance was described as a feasible opportunity to start exercising as a basis for the transition to independent exercise. They described developing knowledge and thinking about exercise during the intervention enabling them to manage the transition to independent exercise. Finding one’s own way for exercise became important for sustaining independent exercise. Lastly, barriers for exercise and strategies for overcoming these were described. Reduced physical health, both temporary and permanent, was described as a considerable barrier for exercise.

**Conclusion:**

The participants described several aspects of participating in exercise that influenced and facilitated their transition to independent exercise. The exercise was experienced as manageable and positive, by a careful introduction and development of an individual exercise routine in partnership with a physiotherapist. This seems to have favored the development of self-efficacy, with importance for future independent exercise. Reduced physical health, both temporary and permanent, was described as a considerable barrier for exercise. The personal process of trying to make the exercise one’s own, and developing knowledge about exercise and new thoughts about oneself, seemed to prepare the participants for managing independent exercise and overcoming barriers.

## Background

Exercise is commonly known to enhance health in all ages and the World Health Organization recommends regular physical activity and exercise to maintain health and prevent disease [1]. Besides disease prevention, exercise also is recommended, with good evidence, as treatment for several chronic conditions [2, 3].

Rheumatoid arthritis (RA) is an inflammatory joint disease that can cause cartilage damage and disability [4]. Today more than 50% of people with RA are above 65 years old [5]. Exercise is suggested as an important part of the management of RA [6, 7], and for older adults the need for disability-preventing physical activity increases with age regardless of diagnosis [8, 9]. However, older adults with RA are less physically active than healthy older adults and do not reach the level of physical activity recommended by international guidelines for health-enhancing physical activity [10, 11]. Several diagnosis-specific barriers for adopting and participating in regular exercise have been reported, such as pain, fatigue, and reduced functional abilities, in addition to general barriers, such as lack of time or motivation [12]. Besides adopting exercise habits, sustaining exercise habits over a long time is known to be challenging and motivation plays an important role in that [13]. Persons with RA partaking in structured exercise interventions have expressed worry about moving on to independent exercise [14], and sustaining independent exercise over time is known to be troublesome for this group of patients [15].

To manage barriers for exercise in older adults with RA, a person-centred approach in the introduction of exercise has been proposed [7]. The person-centred approach emanates from the personal narrative of the person about who she is [16], and the individual is seen as capable and unique [17]. In a person-centred approach, an exercise routine is planned together with the individual, whose resources and wishes are taken in to consideration [16].

There is, to our knowledge, a lack of qualitative studies examining experiences of exercise among older adults with RA. To maintain health benefits from exercise over time it is valuable to increase knowledge about what affects and facilitates the transition to independent exercise. Therefore, the aim of this qualitative study was to explore aspects of participation in moderate- to- high-intensity exercise with person-centered guidance influencing the transition to independent exercise for older adults with RA.

## Methods

A qualitative interview study was performed. Participants were recruited from the intervention arm of a randomized controlled trial evaluating moderate- to- high-intensity exercise intervention with person-centred guidance in 2015–2016 [7]. Recruitment and data collection were performed between January and November 2016. The exercise intervention consisted of 20 weeks of aerobic and resistance exercise at moderate- to- high-intensity. The intervention was led by a physiotherapist according to a person-centred approach, in which the narrative of each person was important for jointly, in partnership, developing an individual exercise routine tailored to that person’s limitations and goals. Through person-centred guidance, the exercise was continuously progressed and adapted to the needs of each person. Exercise was performed according to an individually tailored program where the participants attended a gym at a time of day of their choosing. Several participants attended the gym at the same time, resembling a group session. During 7 months following the intervention, the participants were encouraged to sustain their exercise routine independently and the physiotherapist offered telephone support one to two times during that time period. The intervention had been conducted in three consecutive groups, at two different sites with different intervention leaders, one of which was the first author. Participants were recruited from all three study groups.

Inclusion criteria were: RA according to the American College of Rheumatology 1987 [18] or European League Against Rheumatism 2010 criteria [19], age ≥ 65 years, disease duration > 2 years, DAS28 (Disease activity score 28 joints) below 5.1 indicating a low-to-moderate disease activity (DAS28 range 2.0–10.0), and participation in the underlying exercise study. Exclusion criteria were: ongoing exercise of moderate-to-high intensity ≥2 times/week prior to the start of the exercise study, and inability to understand or speak Swedish.

Participants were invited to participate in this interview study consecutively for each study group after having completed all assessments, performance based tests, patient reported outcome measures and blood sampling both post-intervention and at follow up 7 months after the intervention. The participants were invited face-to-face by a person not involved in interviewing, analyzing or drafting of the current study. The interviews were performed approximately 7 months after the exercise intervention, shortly after the follow up assessment. Sixteen persons were invited to participate and none of the invited persons declined participation. Since the objective was quite narrow, 16 persons were considered sufficient. The material resulting from the interviews is extensive and rich on nuances, which indicates that the number of interviews was enough for saturation. Demographic characteristics of the participants are described in Table 1. Their mean age was 70.6 years, 11 of 16 was female which is approximately the same proportion as in the population, 8 of 16 had a disease activity in remission and the rest had a low-to-moderate disease activity.

**Table 1:** Participant characteristics

Identification for interview	Age	Gender	Disease duration	DAS 28	Co-habiting with an adult	Attendance rate
A	75	Female	14	1.2	no	83%
B	68	Female	8	2.75	yes	83%
C	70	Female	14	3.3	yes	63%
D	76	Female	19	3.48	no	58%
E	75	Male	23	3.56	yes	77%
F	71	Female	18	2.78	yes	88%
G	71	Male	4	3.81	no	65%
H	75	Female	14	3.13	yes	91%
I	71	Female	13	2.36	yes	92%
J	69	Female	≈45	0.76	yes	70%
K	67	Male	4	0.76	yes	75%
L	69	Female	5	1.94	no	82%
M	67	Male	9	2.26	yes	95%
N	68	Female	20	1.74	no	90%
O	68	Male	3	0.49	yes	75%
P	70	Female	10	3.25	yes	65%
Mean/proportion	70.6	11/16	14	2.35	11/16	78%

Age and disease duration expressed in years at the time of interview

DAS28: Disease Activity Score 28, from the time of entering the exercise study. Moderate disease activity > 3.2 > low disease activity. In remission < 2.6

Proportions are presented as women/total and “co-habiting with an adult”/total

### Data collection

Data were collected through qualitative, semi-structured, individual, in-depth interviews. The interviews were conducted by the second author, with experience in qualitative research, who had not been involved in the underlying exercise study. The interviewer had no previous relationship to the participants and were introduced to them as a physiotherapist. An interview guide was developed by the authors, containing open questions about experiences of participating in exercise with person-centered guidance, thoughts about exercise when having RA, and experiences of the transition to independent exercise. A pilot interview was conducted with an older adult with RA with experience of exercise, and did not result in any changes to the interview guide. Each interview was a single session performed in private at the rheumatology clinic, lasting approximately 45–60 min. The interviews were audio recorded and transcribed verbatim. All the transcribed interviews together became the unit of analysis. Field notes was not used in the analysis.

### Data analysis

**Fig. 1:**
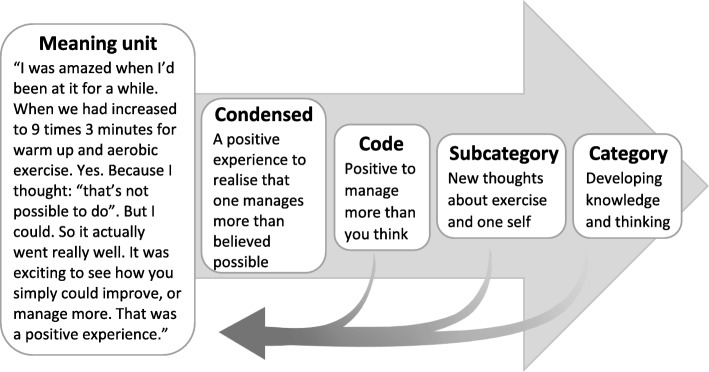
Example of the analysis

Data were analysed using qualitative content analysis [20]. The analysis was performed by the first two authors in collaboration and was validated by the last author, experienced in qualitative research. The interviews were read repeatedly to obtain a sense of the whole. Meaning units were identified based on the objective, condensed, and coded. In the subsequent steps, the meaning units were developed into categories and subcategories (Table 2) with results derived from data. Both manifest and latent content were sought; the manifest content being close to data and the latent content being more abstract descriptions [20, 21]. The analysis moved continuously between the parts and the whole text [20]. Fig. 1 shows an example of the analysis. In the final stage of the analysis process, the authors discussed the categories and subcategories and agreed on the result.

**Table 2:** Aspects of participation in moderate- to high intensity exercise with person-centered guidance that influence the transition to independent exercise for older adults with RA, presented as categories and subcategories

Category	Subcategory
A feasible opportunity to adopt exercise	Person-centred introduction, advancement and adjustments made the exercise feasible
	A new experience of exercise
	A fortunate opportunity to start exercising
	Exercise feasible despite negative elements
Experiencing positive effects of exercise	Positive effects manifested both physically and mentally
	Positive effects manifested in everyday life
	A positive, fun and healthy experience
Contextual factors affect the experience of exercise	Guidance as a security and a driving force
	The possibilities and inadequacies of a loosely connected group
	The facilities influence the experience
Developing knowledge and thinking	Learning about exercise
	New thoughts about exercise and oneself
	The gym as a new arena
Finding one’s way	Fitting exercise into everyday life
	Making the protocol your own
	Transition to independent exercise
	Using tools for motivation
	Access to support
Managing barriers for exercise	Facing barriers for exercise
	Various strategies to overcome barriers
	The importance of determination

Three of the authors are physiotherapists and one is a rheumatologist, three are PhD and all are female. The first author had been engaged as intervention leader in the underlying exercise study which could result in bias but also give valuable insight on the intervention. An attempt to bridle the preunderstanding of the authors was strived for, where the preunderstanding was discussed and made visible and a consciousness about how the preunderstanding could intertwine with the analysis was balanced against a curiosity for the expressed experiences of the interview persons [22]. Quotes are given to support that the results are derived from data. For systematic reporting of the methods section the Consolidated criteria for reporting qualitative research [23] was used.

## Results

The analysis resulted in six main categories and 21 subcategories, presented in Table 2. The exercise with person-centred guidance was experienced as a feasible opportunity to start exercising. The transition to independent exercise was further influenced by a positive experience of exercise, contextual factors, developing knowledge and thinking, finding one’s way and managing barriers for exercise.

### A feasible opportunity to adopt exercise

#### Person-centred introduction, advancement and adjustments made the exercise feasible

When the participants described the development of their exercise routine they talked about how the routine was formed based on their individual abilities, needs and wishes. The person-centred introduction, starting at low intensity with careful progression, was described as enabling their adoption of exercise, despite limitations related to consequences of their disease, such as joint prostheses, cardiovascular disease, or other reasons for low physical capacity. Some participants described how the low intensity introduction was restraining, but that they came to understand the purpose of a light start. The progression of the exercise was also described as positive as it demonstrated increased capacity.

“I got to exercise based on what I could manage, you could say. But anyhow, I got… when I had gotten started, the opportunity to try different things, so to say. It wasn’t just for me to do certain things, but I could add or try what worked for me, and that felt good for me. I thought that was good.” -C.

#### A new experience of exercise

In contrast to the experience of exercise as manageable, several participants described how previous attempts to exercise at gym facilities had failed to succeed due to lack of introduction or structure. The exercise was also described as being at higher intensity than in their previous experiences.

“I have tried, many years ago, but I thought it was really tough with those machines because you never got to learn. Because they left; they showed you ‘it works like this’ and then you were alone the next time.” –L.

#### A fortunate opportunity to start exercising

The participants described themselves as fortunate to have been invited to exercise. The invitation gave them a push to start exercising. Some described the opportunity to participate in moderate- to high-intensity exercise with person-centered guidance as the answer to a personal need. It was also considered interesting to see if any effects would occur.

“I had very poor function in my leg muscles and all. It was necessary…this [the exercise]… I’m so happy they called because I would never have gotten to it myself. So it was pure… it was just like a gift from heaven. I’m very happy about it.” –H.

#### Exercise feasible despite negative elements

Some participants mentioned that exercising could be tough or boring, but yet they wanted to, and managed to, carry out the intervention and sustain independent exercise afterwards.

“I can’t say that it’s super fun to exercise at all times [laughter]. I would maybe rather do something else. But afterwards, I feel so much better.”–B.

### Experiencing positive effects of exercise

#### Positive effects manifested both physically and mentally

Positive effects on body function were experienced by the participants, both physically and mentally. Improved strength, aerobic capacity, mobility, sleep, energy, and mood were described.

“I have become stronger both physically and mentally. Both in getting out meeting people, and feeling that the body works. It means a lot. It does.” -E.

Experiencing positive effects and well-being related to exercise was perceived as increasing motivation for continued, independent exercise.

#### Positive effects manifested in everyday life

The participants also described experiencing positive effects in everyday life, and activities were made possible or became easier. Effects of exercise were also acknowledged by family members and friends.

“When we started here… I couldn’t walk down a stair. But after 5–6 weeks… at home where I live there are five steps to the front door. One day I was in a hurry, and just went straight out. So I wondered ‘What did I do?’ I had to go back and do it again. [Laughter] I could walk down a stair!” –E.

#### A positive, fun and healthy experience

The participants described positive experiences of participating in exercise and the exercise was perceived as fun. Adopting exercise also led to a more positive feeling of other parts of life and a feeling of health.

“I think it’s fun. It’s… it gives a kick for life when you think it’s fun. So that’s really important, to have a positive view on life. So it… It feels good.” –F.

### Contextual factors affect the experience of exercise

#### Guidance as a security and a driving force

The participants perceived the physiotherapist leading the exercise as a security. The presence and competence of the physiotherapist ensured the quality of the performed exercise, and there were always opportunities to ask questions and receive help. The physiotherapist also acknowledged the individual and met individual needs and wishes.

“I think that the physiotherapist was very supportive and encouraging, and pushing at the same time. She told me how it should be and what I should try… and it felt safe. That’s how I would describe the physiotherapist. I felt that she had the situation in her hand in some way.” -A.

However, a few participants said that they were not in need of all the support offered by the physiotherapist during the intervention, or that the need for support was reduced with time.

The physiotherapist was described as a driving force as she pushed the participants to deal with the strain of heavy exercise. The presence of the physiotherapist also gave a feeling of control that prevented deviation from the protocol.

#### The possibilities and inadequacies of a loosely connected group

The participants expressed very different experiences of being part of a group. For some, participating in a group was a positive, social experience that contributed to motivation for exercise and encouraged harder work. That the group members shared the same diagnosis and were approximately the same age was described as positive.

“And we talked about everything except diseases and problems. We talked about good food, good wine… possible acquaintances, mutual acquaintances and that sort of stuff. But we didn’t talk about diseases, our group that was here. It was great… we just noted when someone new came: ‘So you’re also… rheumatic.’ And that was that.” -E.

Others did not experience any connectedness to a group at all, which was a drawback for some and a benefit for others depending on differing social needs and longings.

“I thought it was quite good, that you didn’t need to adjust to anyone, someone who doesn’t keep up or so. If you’re a group where everybody is supposed do the same thing at the same time, and there is someone, or it might be yourself, who don’t keep up. That’s not particularly fun. That’s why I thought it was quite good that you could exercise on your own.” –D.

#### The facilities influence the experience

There was a wide range of experiences of the gym facilities among the participants. Some found the facilities to be a negative experience mostly due to music volume and location. Others appreciated the facilities and struggled to find a similar alternative. A nearby geographic location was seen as a strength when choosing a facility. Other important factors were the cost, the equipment of the facility, the air quality, and the environment of the facility.

“The question is if I should keep going to [the intervention gym]. I have to go by car since there is a distance. Or should I find another gym? At [the intervention gym] there were space and there were always people, it was clean and tidy, lockers and so, clean and tidy in the showers and it felt great. I have visited another gym and I can’t imagine going there, it felt… no. So that matters, at least to me, how it looks at the gym.” –C.

### Developing knowledge and thinking

#### Learning about exercise

Through the person-centred guidance the participants described gaining knowledge about exercise management and aerobic and resistance exercise at high intensity. This new knowledge was used to adjust their independent exercise routines, both when resuming exercise after a break and when in need of increased exercise loads.

“Since the exercise was new for me it was even more important to learn the movements. And when I came to [a gym facility near home] there were the same machines and the movements were still the same.” -P.

#### New thoughts about exercise and oneself

The participants described how their thoughts about exercise changed due to the performed exercise. Some stated that their views on intensity and the need for regularity of exercise had changed. Some stated that they had learned that exercise was not dangerous despite pain and disease. They also recognised that they were capable of more than they first thought.

“Now I know that I can exercise just as much as everyone else. I don’t need to be afraid to go to the gym and I can just leave out certain things if I realise that I can’t handle them.” -N.

Some also described how the onset of exercise also brought other personal changes, such as a higher priority of one self or smoking cessation.

#### The gym as a new arena

The participants described how they overcame a previous sense of resistance towards the gym environment. However, someone still described the gym as not for them after the moderate- to high-intensity exercise with person-centred guidance. The process of becoming familiar with a new facility was by some participants described as taking some time and by others as being unproblematic.

“…it’s very fun now to be a person that knows what it means to exercise, and suddenly I meet lots of friends that also exercise. Yes, you feel that you are part of a contemporary movement, a contemporary phenomenon.” -N.

### Finding one’s way

#### Fitting exercise into everyday life

The participants described strategies and important factors for fitting exercise into their everyday life, both during the intervention and during the follow up period. For a limited time a more extensive expenditure of time was reasonable but to incorporate exercise in the long term two times a week was described as reasonable. However, both one and three times was also mentioned as reasonable for some and some mentioned that as a pensioner there is time for exercise.

“You have to have a life outside this as well. So I thought this was perfect. And it took about one… one and a half hour. That was just right. I think that she [the physiotherapist] had figured that out just right.” -L.

The flexible time for exercising facilitated the development of a personal arrangement for exercise, since the preferences differed among individuals. Some described that the development of exercise routines formed habits and created a need for exercise. Everyday walks and other types of exercise were also described as part of a new routine.

Several of the participants described a wish and a need for seasonality of exercising. The seasonality could be related to a wish for exercising outdoors in the summer and for a change of routines related to summer homes and gardening.

#### Making the protocol your own

The participants described the process of making the exercise protocol ‘their own’ and adopting it as a personal routine. The descriptions of the practicalities in forming their own protocol varied from strictly adhering to the initial exercise protocol, to finding their own programme by adding other kinds of exercise activities to their weekly routine. The participants also described how they modified the protocol in terms of repetitions and sets at the gym.

“I had planned to quit exercising after this, but I did the tests before, and after five months, and my results were apparently really good, according to the doctor. So I thought ‘I be damned if I quit’, so I kept exercising. I exercised at the gym until my membership ended, then I went swimming, and later I started to run with my dog, up to five times a week.” -K.

#### Transition to independent exercise

Many of the participants perceived that the step to independent exercise was unproblematic, because they could stick to established routines from the intervention. Someone described that it helped to already be familiar with the facilities. Someone speculated that it would have been easier to continue at a new facility if you were accompanied by a friend. However, a few participants described how they did not manage to go through with the step to independent exercise and stopped exercising.

“It was no problem, no problem at all, I just kept going. So it… it was no problem at all, it went well.” –C.

#### Using tools for motivation

Different tools for increasing motivation for exercise were described. The exercise diary used during the intervention period was described as a helpful tool that several participants continued to use independently. At the same time, the diary was described as unappreciated by others who were not motivated to keep a diary during or after the initial period. A social element helped several participants keep motivation as they perceived it as meaningful to meet friends or acquaintances at the gym. Another important motivational factor for some was having long term goals related to everyday life.

“My goal is to do what I can myself to influence my possibilities to stay in my current home for another 20 years, and take care of myself. I want to be able to carry my wine bags on Fridays for example, and grocery bags. I’m happy to accept help if someone drops by but I want to be able to do it. That’s what motivates me, I would say.” -A.

#### Access to support

The participants described various ways of receiving support for exercise during the follow up period. Some had positive experiences from continued coaching, through for example professional introduction at a new facility or through appointments with personal trainers. Some sought help from a physiotherapist in the healthcare system. Some participants considered the possibility to get help but had not used that possibility yet and some had not seen a need for further coaching.

Some participants also described missing the continuous contact with the physiotherapist in their independent exercise. Telephone calls from the physiotherapist during the follow-up period instilled a sense of being cared for, and was perceived as support for continued independent exercise. The phone calls also provided a feeling of being supervised, which increased motivation for independent exercise. Some described the feeling of being controlled as unwanted and declined follow-up phone calls.

“She [the physiotherapist] asked if we wanted to [be phoned]. And I said ‘that’s good’ because then you still have some… motivation and so, to not quit immediately.” -J.

### Managing barriers for exercise

#### Facing barriers for exercise

Reduced physical health, ranging from light infections to chronic diseases and hospitalisation, was perceived as a barrier for exercise. Other barriers were reduced motivation without coaching, economic factors, poor sleep, and competing activities. However, some participants perceived no barriers during the follow up period.

“It wasn’t a conscious strategy of mine, to refrain from exercise. That’s not it. It’s rather the body talking, or my health status talking. I can’t. I would’ve liked to go there anyway, but I can’t.” -D.

#### Various strategies to overcome barriers

The participants also described their strategies to overcome barriers. These could be mental strategies, such as keeping to the routines; physical strategies, such as using orthoses; and social strategies, such as contacting a trainer.

“I was furious. Totally furious. I was… I thought I was doing so well. And the foot was swollen, I couldn’t get my shoes on, I couldn’t walk and I couldn’t do anything. But then I thought ‘now I just have to accept this’. Even my husband said that if I hadn’t been exercising he wouldn’t have wanted to be at home with me, since then I wouldn’t have managed at all. And I thought ‘Maybe I could do a little exercise.’ And I did exercise with my arms and hands. Otherwise I would have been sitting there longer.” -B.

#### The importance of determination

Participants who had encountered barriers talked about a strive to get back to exercise, and having more or less detailed plans. Others also described the importance of being determined to exercise to be able to overcome barriers.

“I could do it all. But the thing is…. You can’t do it if you don’t want to. Many times when the will is lacking, you have to tell yourself that you can do it. And then you can and in the end it’s easy.” -F.

## Discussion

The results of the study described aspects of participation in moderate- to high-intensity exercise with person-centred guidance that influenced the transition to independent exercise. Older adults with RA described experiences that where both facilitating and hindering.

The person-centred introduction to exercising at low intensity, followed by careful progression, might have contributed to making the exercise manageable by avoiding pain and physical problems related to the joint disease. The introduction also appeared to support performance accomplishment which is important for recognising personal abilities and for developing self-efficacy [24]. Perceived self-efficacy refers to a person’s beliefs about her capability to perform in certain events and is important for thinking, behavior and motivating oneself. A stronger sense of efficacy facilitates performance accomplishment and well-being and at the same time could be developed through personal experience of mastery, of seeing others succeed and through persuasion [25]. To expect a certain outcome is important for motivation [25] and experiencing positive effects of the exercise, both physically and mentally, appears to have contributed to the participants’ overall positive experience of exercise also increasing their self-efficacy for management of independent exercise in new settings. Experienced positive effects, also acknowledged by others, could be viewed as confirmation of the effectiveness of the intervention. Increased knowledge, through experience, of the effects of exercise has been suggested to add to motivation for exercise [14].

In addition to increased knowledge, the participants described how their thoughts about exercise and about themselves, as someone exercising with a diagnosis, had developed. This new way of thinking about oneself and a revised belief of what is possible can be interpreted as increased self-efficacy [26]. When the participants expressed that they had entered the gym as a new arena, it might be a response to a new confidence in their ability to exercise and a feeling of security in an environment to which they might have previously been unaccustomed. Developing self-efficacy during an exercise intervention seems to be important for sustaining independent exercise [27] and it could have an even more important role as mediator for exercise in older adults where age-related physical decline is present [28].

The results of the study suggest that finding your own personal way of exercising is important both for adopting exercise and sustaining independent exercise habits. Besides practicalities in developing the protocol and fitting exercise into everyday life, the participants also described a mental process of making the protocol their own. The participants often developed an individual idea about what amount of time that was reasonable to set aside for exercise. Some developed strict routines for their independent exercise to be able to coordinate exercise with other activities, whereas others valued flexibility in when to perform their independent exercise for the same reason. Flexibility and independence have been mentioned as one of the benefits of home exercise [29], but our study suggests this to be achievable also in exercising at a facility under the right conditions. Although flexibility provides a certain freedom, the establishment of routines has been described as important for maintenance of exercise [30]. Keeping to the routines was described in this study as one strategy to overcome different barriers.

One considerable barrier for exercise was perceived to be reduced physical health. Because this is a known barrier both for persons with RA [29] and for older persons [31], the results of this study could be interpreted as physical health being an even more important barrier to address for older persons with this type of chronic disease. In order to recover from this barrier and to manage independent exercise there could be an increased need for temporary support.

Different participants had their experience of exercise affected by contextual factors in various ways. It is interesting to note that there was a rich diversity in experiences of the same factors among the participants. In view of this diversity, the person-centred approach might be helpful to understand and adjust these different contextual factors to fit the individual’s needs and wishes, in order to facilitate the transition to independent exercise.

The moderate- to high-intensity exercise was performed in a loosely connected group, and the participants described both possibilities and inadequacies related to that structure. The participants described the benefits of a diagnosis-specific group, where the diagnosis itself shifts from being a factor that differentiates the individual from others to a factor common with others. Ricoeur [17] describe likeness and unlikeness at the same time as specifically human, as one needs to be open for differences in other people and confirm these differences in order to confirm oneself. The diagnosis-specific group enabled the participants to be confirmed as individuals and humans based on their unique qualities other than the diagnosis, because the diagnosis was part of the sameness in this context. Sharing experiences in a diagnosis-specific group emphasises understanding of each other’s symptoms [32], and has been described as being part of a community [33].

In addition to the benefits of sharing a diagnosis, the participants in this study also highlighted the benefits of being of the same age. The social interaction of persons of the same age is particularly important in providing a sense of security [34] and groups too heterogeneous regarding age have been described as problematic [14]. Socialisation itself may also encourage physical activity [35]. Comparisons during exercise may motivate a higher exercise effort and could also stimulate peer learning [29], while watching someone with the same attributes as oneself succeed with a task, may increase self-efficacy [24, 25].

The participants of this study described different needs and expectations of being part of a group. As a professional it is important to know that some individuals decline any form of group participation while others prefer a facilitated group introduction. Many participants acknowledged that being part of a group might not be for everyone; a finding that has previously been reported [35]. The design of the exercise intervention in the current study, which allowed participants to perform individual exercise collaterally, provided a good opportunity to either seek or reject contact with other group members.

The participants described the physiotherapist as providing security and at the same time being a pushing driving force. The person-centred approach might be a key factor in managing the balance between being a security offering help, and pushing to greater achievements and supporting independence. The person-centred approach entails an establishment of a partnership between the patient and the professional [16] which is desired since patients have been reported to want to be seen as experienced experts in the partnership for managing a rheumatic disease [36]. In addition to the value of a personal partnership, the competence of the person recommending exercise seems to be important [35], which aligns with this study’s expressions of competence as an assurance for quality and safety in exercise. The support from a physiotherapist as a knowledgeable professional has previously been described as facilitating the step to independent exercise [29].

### Strengths and limitations

This is, to our knowledge, the first study exploring the influence of aspects of exercise with a moderate to high intensity, among older adults with RA, on the transition to independent exercise. A qualitative research design is appropriate for this type of objective. Qualitative content analysis is a commonly used analysis method in health care [32, 37].

A limitation of the current study is that all participants were recruited from the same, specific exercise intervention and their experiences might not be transferable [21] to other types of exercise interventions. To enhance credibility, participants with various experiences were sought by recruiting from all three study groups and from both genders [20]. Among the recruited participants there was a diversity in experiences of continued exercise representing both successful transitions and discontinued exercise. In this cohort all participants were recruited with a disease activity below DAS28 5.1 and 8 out of 16 had a disease activity in remission. This means that this results cannot be assumed to be transferable to older adults with a high disease activity. At the same time a low disease activity is nowadays common among patients with RA due to the advances made in pharmacological treatment [38].

It has been argued that pure induction is not possible in qualitative content analysis and the analysis process has been described as to some extent moving between an inductive and deductive approach, not least because of the preunderstanding of the authors [21, 39]. As reflexivity is of importance for the quality of this work [40], the analysis and presentation was questioned and the preunderstanding of all authors, and its role in the drafting and performing of the study, was discussed. Bridling of the preunderstanding was strived for in the analysis. As the first author was involved both in the underlying exercise study and in the analysis, parallel analysis by several authors was performed to address the issue of dependability [21]. All authors were in agreement on the final result.

The Consolidated criteria for reporting qualitative research criterion of member checking was not fulfilled and the value of this process as validation has been discussed [41]. That could mean that participants has been misinterpreted but editing of data due to such checking could also be considered a data event.

### Implications

This study adds understanding about different aspects of participating in exercise that could be important to take into account when aiming at facilitating independent exercise for older adults with RA. Several aspects concern feasibility of this design of moderate- to high-intensity exercise with person-centred guidance and supports that it could be useable in a clinical setting. However, the finding that physical health was a considerable barrier highlights the importance of addressing this barrier to a greater extent for older persons with this type of chronic disease.

## Conclusion

The participants in this study described several aspects of participating in exercise that influenced and facilitated their transition to independent exercise. The demanding exercise was experienced as manageable and positive despite their age and diagnosis, by careful introduction and development of an individual exercise routine in partnership with a physiotherapist. This seems to have favored the development of self-efficacy, which had importance for future independent exercise. Reduced physical health, both temporary and permanent, was described as a considerable barrier for exercise. The personal process of trying to make the exercise one’s own, and developing knowledge about exercise and new thoughts about oneself, seemed to prepare the participants for managing independent exercise and overcoming barriers.

## Abbreviations

DAS28: Disease activity score 28
RA: Rheumatoid arthritis
